# Molecular analysis of bacterial diversity in mudflats along the salinity gradient of an acidified tropical Bornean estuary (South East Asia)

**DOI:** 10.1186/2046-9063-10-10

**Published:** 2014-10-30

**Authors:** Henk Bolhuis, Henriette Schluepmann, Juri Kristalijn, Zohrah Sulaiman, David J Marshall

**Affiliations:** 1 Department of Marine Microbiology, Royal Netherlands Institute of Sea Research (NIOZ), P.O. Box 140, 4400AC Yerseke, The Netherlands; 2 Molecular Plant Physiology, Utrecht University, Padualaan 8, 3584CH Utrecht, The Netherlands; 3 Environmental and Life Sciences, Faculty of Science, Universiti Brunei Darussalam, Tungku Link, Gadong BE1410, Brunei Darussalam; 4 Institut Teknologi Brunei, Tungku Link, Gadong BE1410, Brunei Darussalam

**Keywords:** Marine Environmental Research, Borneo, Brunei, Mudflats, Acid sulfide, 16S rRNA, Bacterial diversity, Microbial mats

## Abstract

**Background:**

The Brunei River and Bay estuarine system (BES) in the northwest of Borneo is acidic and highly turbid. The system supports extensive intertidal mudflats and presents a potentially steep salinity and pH gradient along its length (45 km). Temporal variation in physical parameters is observed diurnally due to seawater flux during tidal forcing, and stochastically due to elevated freshwater inflow after rains, resulting in a salinity range between 0 and 34 psu. High velocity freshwater run-off from acid sulphate formations during monsoon seasons results in highly variable and acidic conditions (pH 4) at the upper reaches of the BES, whereas the pH is relatively stable (pH 8) at the seaward extremes, due to mixing with seawater from the South China Sea. At their surfaces, the BES mudflats present microbial ecosystems driven by oxygenic phototrophs. To study the effect of various physical parameters on the bacterial diversity of the BES mudflats, surface samples were collected from six sites stretching over 40 km for molecular and phylogentic analysis.

**Results:**

The bacterial diversity at these sites was compared by community fingerprinting analysis using 16S rRNA gene based denaturing gradient gel electrophoresis and by 16S rRNA gene sequencing and phylogenetic analyses. Results revealed functionally conserved, diatom-driven microbial mudflat communities composed of mainly novel, uncultured species. Species composition was evaluated as 50-70% unique for each site along the BES. Clustering of the sequences commonly occurred and revealed that proteobacterial diversity was related to the salinity gradient. When considering all phyla, the diversity varied consistently with physical parameters (including anthropogenic) that are expected to influence microbial composition.

**Conclusion:**

The BES mudflats were found to comprise the typical functional groups of microorganisms associated with photosynthetic carbon flux, sulfur cycling (Gamma- and Deltaproteobacteria), and decomposition (Bacteroidetes). From a structural perspective, however, the mudflats constituted discretely distributed communities along the physical gradient of the BES, composed of largely novel species of Bacteria. This study provides first insights into patterns of bacterial community structure in tropical South East Asian coastal ecosystems that are potentially threatened by increasing variability in pH and salinity, in line with predicted future environmental change.

## Background

Mudflats are found all over the world in intertidal and freshwater systems. They possess an enormous water carrying capacity and therefore serve as natural coastal defence systems. They can be extremely productive and may be responsible for up to 50% of the primary production of estuaries, and thereby sustain large fish and shellfish stocks [1]. Diatoms are photosynthetic microalgae that form extensive biofilms or mats on the surface of intertidal mudflats providing a matrix in which the organisms are embedded. Diatoms are primary producers, forming large amounts of extracellular polymeric substances (EPS). These substances play an important role in sediment stabilization [2,3], but also provide an important input of organic carbon that supports a complex ecosystem, consisting of several trophic levels of organisms, ranging from macro- to micro-fauna [4]. In these estuarine environments, anoxic conditions in mudflats rapidly evolve and stimulate sulfate-reducing prokaryotes that use sulfate as the terminal electron acceptor and produce large amounts of sulfide [5]. A part of the sulfide is converted back to sulfate by sulphide-oxidizing bacteria, thus completing the sulfur cycle. A greater part of the sulfide, however, remains bound by reactive metal ions, and forms insoluble metallic precipitates, principally minerals containing FeS and FeS_2_ (iron pyrite) [5,6]. The bacterial sulfur cycle is largely maintained by photosynthetically produced carbon and energy sources, mainly sugars and low molecular weight organic acids such as acetate, lactate and sulfonates [7-9]. Sulfate reduction is considered as one of the main anaerobic processes in the biomineralization of up to 50% of the total organic matter in mudflat sediments [10]. The majority of studies on microbial diversity and functioning of mudflats has been undertaken in temperate regions [11-14]. Although much less is known about microbial communities in mudflats from tropical regions, information is becoming available for the subtropical Asian region [15,16].

**Figure 1:**
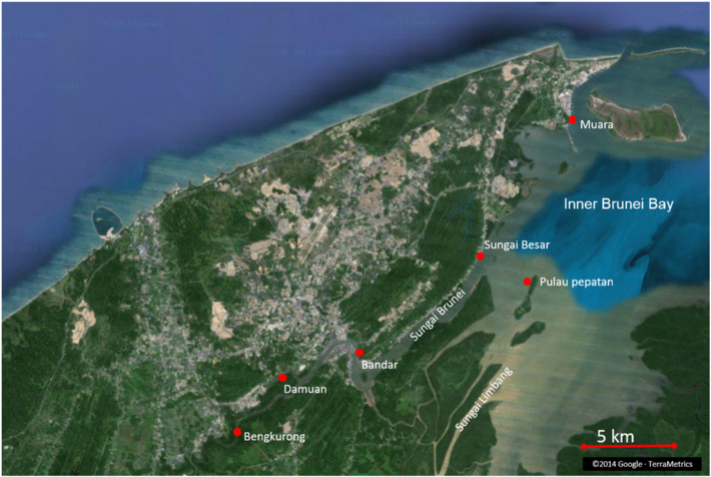
**Map of the Brunei River and Estuary System.** Sampling points are indicated by red dots. Shallow areas in the inner Brunei Bay (depth ~5 m) seen as brown. Daily tidal range varies between 0–2.3 m. Relatively high turbid inflow is shown associated with Limbang River. Blue areas show the distribution of deeper channels.

In the tropical Brunei River and Bay estuarine system (BES) of the South East Asian island of Borneo (Figure 1), mudflat inundation is highly variable in duration and the water may vary considerably in its physical and chemical properties. The mudflats experience seasonally-changing, daily semidiurnal tidal patterns, and seasonally-variable freshwater inflow associated with monsoon and inter-monsoon periods (two of each annually). At times the mudflats are exposed to desiccating conditions. Importantly, mudflats in the BES are strongly influenced by acidic freshwater running from alluvial deposits dating 5400 years ago in the valleys and older sediments on the surrounding terraces made up of acid sulfide containing soils with little to no carbonate-containing mineral to neutralize [17-19]. These acidic freshwater inflows become mixed with more basic marine water along the length of the BES. Sulfide minerals may result from the above described bacterial process occurring in the geological past as is the case for the Setap Shale in Brunei [20]. When exposed to aerobic environments by erosion or excavation, sulfide minerals will oxidize and eventually leach sulfuric acid which, if not neutralized, may cause soil pH to drop to pH 3.5 or lower, and aluminium or heavy metals to be released [17,21-23]. Moreover, as in many estuaries, water runoff into the BES is influenced by a muddy substratum, causing turbidity that limits photosynthesis, thus limiting active withdrawal of CO_2_ and reducing the production of O_2_[24]. Organisms inhabiting the naturally acidic BES are thus exposed to pH and salinity levels ranging beyond 5.8 and 8.3 pH units and 3.58 and 31.2 psu [25], but also to high natural (mangrove) and anthropogenic organic loads, low dissolved oxygen levels, elevated dissolved CO_2_ levels, and highly turbid conditions [26].

Microbial communities along natural salinity and pH gradients in estuaries are generally believed to play an important role in ecosystem stability [27-30]. The BES presents the opportunity to explore the microbial community composition for a tropical estuarine system, strongly influenced by acidic inflow [21]. There is heightened interest to understand the effects of acidification on patterns and processes in marine ecosystems in general, in line with the current threat of globally lowered seawater pH, relating to the ongoing elevation of atmospheric CO_2_[31,32]. In the BES, microbial community composition may on one hand be influenced by the physical (pH/salinity) gradient, but microbial activity may also be directly responsible for the observed acidification through fermentation or respiratory redox chains. In the present study we used molecular tools to analyse the bacterial community composition in mudflats along the BES gradient and reveal a community largely consisting of unique species.

## Results

Most of the microbial mats revealed clear photosynthetic activity with large amounts of oxygen being produced at the surface. Mat pigmentation at extraction was more brown than green suggesting the dominance of diatoms over cyanobacteria (data not shown). Only the sediments from the most seaward sampling site at Muara, which had a more sandy structure, did not visibly show oxygen production. Upon sampling all sites revealed the typical odour of sulfides, indicative of an active sulfate reducing community. At the time of sampling, we were unable to obtain accurate measurements of salinity and pH in the sediments. We therefore measured the pH and salinity of the water to which the sediments are exposed (Table 1) at maximally one meter distance from where the sample was taken. For Pulau Pepatan the sampling point was far from the water mass due to low tide and we used a puddle of water adjacent to the sampling spot instead. The salinity of the overlying water at this site is usually highly variable, being influenced by marine water at high tides and by fresh water from the Limbang River and during extensive rainfall. At time of sampling the salinity was rather low (9.3 psu) but recordings as low as 3 psu have been measured before at this site.

**Table 1:** Water variables at sampling points in the BES (04 November 2012)

	**Coordinates**	**Salinity (ppt)**	**pH**
Bengkurong	4°49’09” N 114°51’43” E	3.04	4.60
Damuan	4°52’08” N 114°54’42” E	8.12	7.49
Bandar	4°53’10” N 114°56’50” E	7.57	7.46
Sungai Besar	4°55’40” N 115°00’52” E	15.45	7.95
Muara	5°00’23” N 115°03’57” E	28.0	8.78
Pulau Pepatan	4°53’09” N 115°02’41” E	9.3	8.15

**Figure 2:**
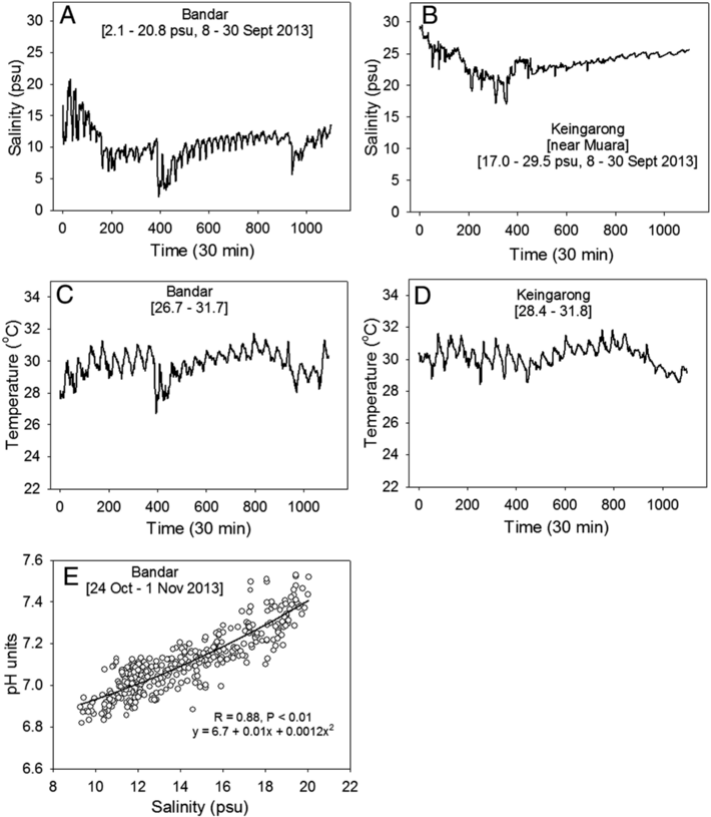
**High resolution salinity and temperature data for the BES estuarine water. A**-**D)** Recordings were made every 30 min, between 8 and 30 September 2013, simultaneously at Bandar and Keingarong (near Muara). Variations in diurnal (tidal) and longer-term measurements of salinity were greater at Bandar compared to Keingaron, and the salinity regime at Keingaron was distinctly higher. Temperature varied in a similar way at each site. **E)** The relationship between pH and salinity for simultaneous recordings (every 30 min) for just under 1 week (24 October to 1 November 2013).

**Figure 3:**
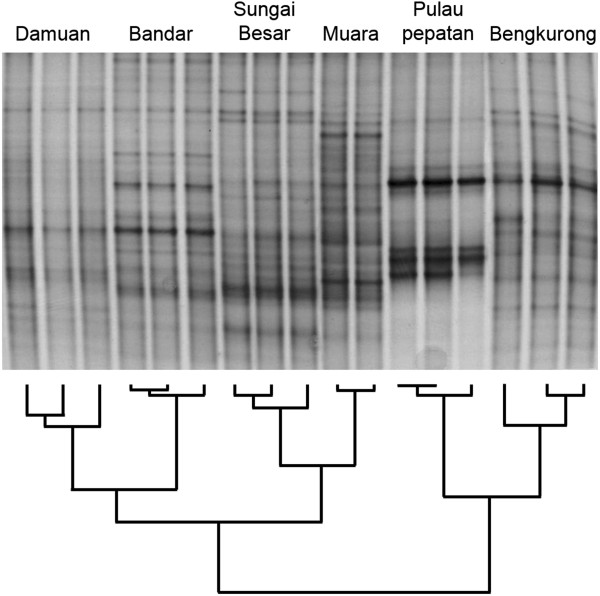
**Bacterial 16S rRNA Denaturing Gradient Gel Electrophoresis analysis.** Samples were obtained and analysed in triplicate. The gel was slightly modified in Photoshop to accommodate the obtained clustering information with BioNumerics software (the three lanes covering the Bengkurong samples were transferred to the right part of the gel).

In accordance with estuary dynamics that are influenced by both freshwater from the river and marine water from the tides, the salinity increased from the upriver sampling point at Bengkurong to the most marine sampling point at Muara (Table 1). These spot recordings were consistent with more detailed high-resolution temporal data recorded simultaneously at Bandar (landward) and Keingarong (5°02’05”N 115°06’02”), near Muara, every 30 minutes, for 22 days (see Figure 2). Both diurnal (tidal) and longer-term salinity variations were greater at Bandar (ranging between 2.1 and 20.8 psu) compared to Keingarong (17.0 and 29.5 psu), for which the salinity regime was distinctly higher. Water temperature was similar among the stations (~30C°), and pH closely correlated with estuarine water salinity (Figure 2).Bacterial 16S rRNA gene DGGE analysis revealed a diverse banding pattern, indicative of a rich bacterial community (Figure 3). The triplicate samples from each location were nearly identical in composition. In contrast, comparison of the samples between locations revealed clear differences in banding pattern, although a number of shared bands were observed in samples from most locations. The mudflat samples from Pepatan Island appeared less diverse with a lower number of visible bands. Cluster analysis of the DGGE fingerprints revealed two major clusters, the first consisting of two sub-clusters containing freshwater-dominated mudflat sites with high organic load Damuan and Bandar, and of the marine-dominated mudflats Sungai Besar and Muara. The upriver site Bengkurong clustered separately with the outlier Pulau Pepatan situated in the BES bay.

**Figure 4:**
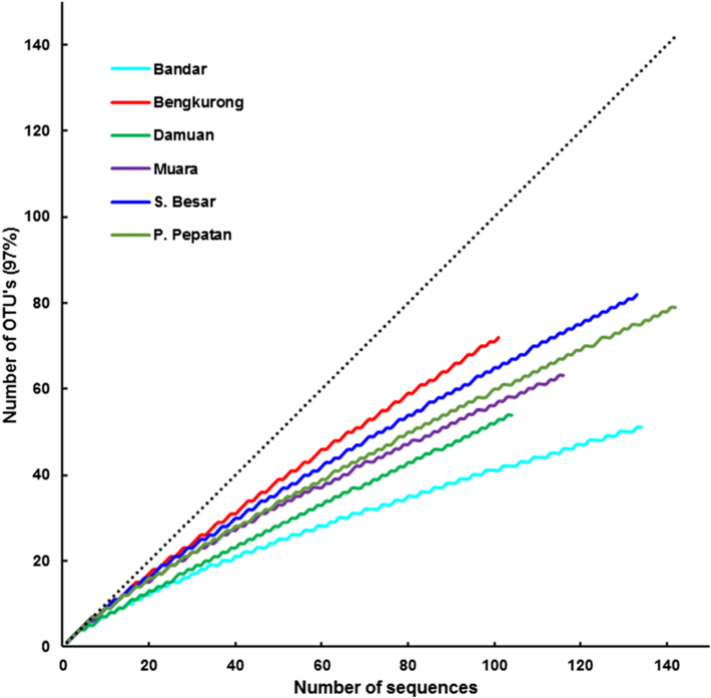
**Rarefaction analysis of the OTU’s of the six samples at 97% sequence identity.** The dotted line indicates the 1:1 line.

Based on the large overlap in community composition revealed by DGGE, DNA extracts from replicate samples in one location were pooled to represent the unseen heterogeneity of the mat type in further molecular analysis. For each location, a 16S rRNA gene clone library was constructed from the pooled samples consisting of over 100 clones with ~ 600 nucleotides per read (Table 2). Richness estimators (Chao) [33] at a 97% identity cut-off revealed the highest values of 386 and 430 operational taxonomic units (OTU’s) for the two upstream freshwater- influenced sediment samples, Bengkurong and Damuan respectively, and the lowest richness of 192 for Bandar. The calculated rarefaction curves for the six samples leveled off from the 1:1 interval but were far off from reaching an asymptote (Figure 4). The estimated richness declined to almost half of the highest estimates towards the marine sites (Table 2). The micro-diversity at 100% OTU cut-off revealed the highest richness in the Sungai Besar samples with 2883 estimated OTU’s (data not shown). The Shannon diversity index calculated at a 97% cut-off value varied between H = 3.1 and H = 3.9 with the highest diversity found in the Bengkurong mudflat samples and the lowest in Bandar and Damuan.

**Table 2:** Diversity and richness indices at 97% cut off value

**Sample**	**# clones**	**Sobs**^**a**^	**Chao**	**Shannon (H)**
Bengkurong	106	72	351	3.9
Damuan	134	54	430	3.1
Bandar	116	51	192	3.1
Sungai Besar	116	82	193	3.7
Muara	133	63	231	3.6
Pulau Pepatan	142	79	237	3.8

^a^ Observed OTU’s.

**Figure 5:**
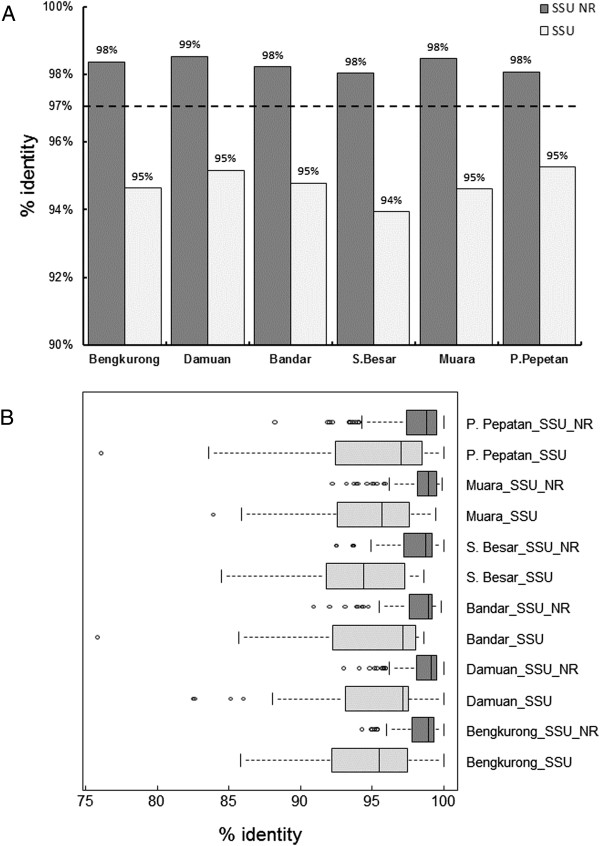
**Clone library sequence identities and percentage novel OTU. A)** Percent identity of clone library sequences with the Silva database of uncultivated species (SSU NR) and of well characterized cultivated species (SSU). **B)** Percentage of novel sequences, defined as sequences with less than 97% sequence identity, presented as a boxplot.

**Figure 6:**
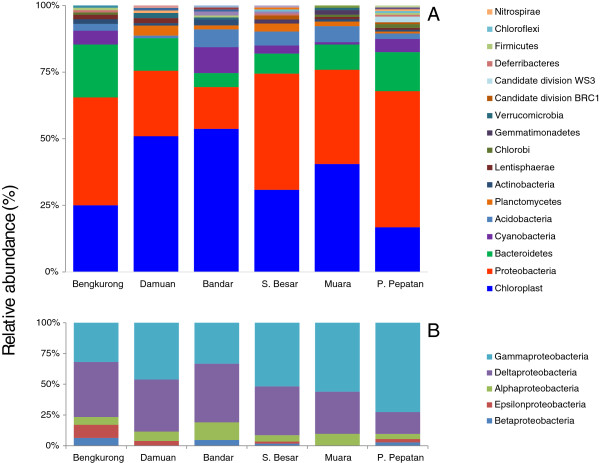
**Taxonomic classification of the obtained 16S rRNA gene sequences. A)** Relative abundance at the phylum level and **B)** detailed abundance of the proteobacterial classes.

**Figure 7:**
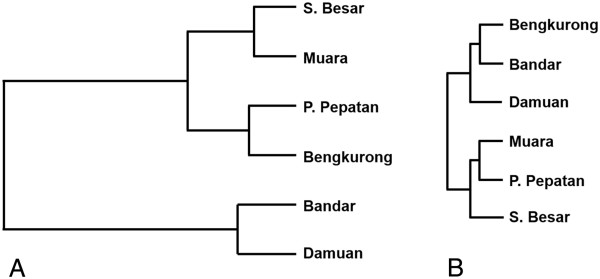
Cluster analysis of the phyla A) and proteobacterial class B) distribution in the six samples.

Comparison of the obtained sequences with the Silva databases revealed that for all samples most sequences only have a high identity (average of 98.3% identity) with uncultivated species from the Silva SSU NR ref database (Figure 5A). The average identity of all sequences with the SSU database of cultivated species is 94.7%. The lowest identity for a sequence read was found in Bandar, this DNA sequence revealed 75.8% identity with a sequence from the SSU database of cultivated species and 96.7% identity with a sequence clone designated as belonging to Candidate divisions BD1-5 and more particularly with a strain identified in hypersaline microbial mats in Guerrero Negro [34]. Further analysis of the identities showed that a large fraction (average of 56.7%) of the sequenced clones was derived from species with less than 97% identity to known isolated species and 17.2 % of the sequences had less than 97% identity with all known sequences in the Silva database. Most novel species were found in Bengkurong and Sungai Besar; respectively 69% and 68% of the clones had less than 97 % identity with the best match in the SSU database versus 18% and 22% with the SSU NR database (Figure 5B). Comparison of the shared number of OTU’s between the samples from different locations using the “summary.shared” command in Mothur revealed that at a cut-off level of 97% on average 90% of the OTU’s in pooled samples from each location is unique to that site (data not shown). The highest number of overlapping sequences with 97% or more identity between two locations was only 6 for Muara and Sungai Besar.Taxonomic annotation of the 16S rRNA sequences showed that Proteobacteria and chloroplasts derived from diatoms form the dominant groups in the Brunei mudflats taking up more than 60% of the total diversity. Chloroplast sequences form the majority in Bandar (53.7%), Damuan (50.9%) and Muara (40.5%) and form the second largest group in Sungai Besar (30.8%), Bengkurong (25%) and Pulau Pepatan (16.8%). With 51%, 43.6% and 40.5% of the total sequences, Proteobacteria dominate the populations in Pulau Pepatan, Sungai Besar and Bengkurong respectively (Figure 6A). These two groups were followed in order of abundance by Bacteroidetes (on average 11%), Cyanobacteria (4%), Acidobacteria (4%), Planctomycetes (~2%) and Actinobacteria (1%). The diversity is further followed by a number of species with only one or two representatives in the clone libraries. Amongst these are a number of sequences derived from candidate divisions several of which still lacking cultivated representatives. These candidate division related sequences are often confined to one or two samples.Further dissection of the proteobacterial phylum revealed that Delta- and Gammaproteobacteria formed the dominant groups making up 80 – 90% of the Proteobacteria and with a trend of more Deltaproteobacteria in the freshwater sites and more Gammaproteobacteria in the marine sites (Figure 6B). Alphaproteobacteria are found in each site at approximately 10% of the total proteobacterial fraction. Beta- and Epsilonproteobacteria are present in some samples and only at low abundance.Cluster analysis of the phylogenetic distribution within the samples show clustering of the freshwater sites Bandar and Damuan and of the marine sites. The most upstream located freshwater site Bengkurong clusters with the marine outlier site Pulau Pepatan (Figure 7A). Clustering of the proteobacterial classes gave a clear distinction between the marine and freshwater communities (Figure 7B).

## Discussion

The Brunei River and Bay estuarine system forms a unique tropical ecosystem that has not yet been the subject of microbial diversity studies. The nearly continuous input of acidic, sediment-rich freshwater in the Brunei Bay provides a unique opportunity to study consequences of acidification and eutrophication on marine ecosystems. For example, Marshall and co-workers showed that acid input from the Brunei River led to a dramatic erosion of gastropod shells [25]. The microbial study presented here focuses on the mudflats along the Brunei River into the bay. Since mudflat sediments are quite stable with respect to pH homeostasis we did not directly expect nor look for changes related to pH but rather focussed on the increased salinity from river to sea. While the column water salinity and pH of the BES were strongly correlated (Figure 2; [25]), preliminary assessments suggest relatively invariable pH in BES sediments (unpublished observations; Hossain, PhD thesis). Salinity is of special interest since it is a well-known driver for microbial diversity and in contrast to pH cannot be regulated by microbial activity [35].

The first noteworthy observation when looking at the OTU statistics is the very low identity with known cultivated bacterial species. The fact that almost 60% of the sequences in our clone library have less than 97% identity with known species shows that the Brunei mudflats harbour a unique undiscovered microbial ecosystem. Bengkurong and Sungai Besar harbour the most novel species, even when compared to sequences obtained by cultivation independent studies that reveal about 20% with less than 97% identity. This is also reflected in the taxonomic identification revealing that 11 out of the 28 identified phyla belong to candidate divisions (BRC1, WS3, TA06, BD1-5, BHI80-139, OP11, OP8, JL-ETNP-Z39,NPL-UPA2, RF3, TM6), most of which still have no cultivated representatives. Although these candidate divisions are only represented by low numbers of sequences in our data set, they still make up 1-2% of the total community and should be considered as abundant rather than rare. Obviously high throughput sequencing will provide a better insight in the true rare microbial diversity [36-38] and is planned for a follow-up analysis on this ecosystem. Even at a lower taxonomic level (order, genus, family), several novel “candidate” taxa are found including species with a yet uncertain taxonomic placement (Class *Incertae Sedis*). For example, amongst the Bacteroidetes we found representatives of group BD2-2, SB-1 and vadinHA17. Most of these candidate divisions and ‘uncertain’ groups are currently identified in a wide and diverse variety of environments making a potential link to ecosystem functioning impossible until members of these groups are isolated and characterized or until full genomic information is available. Despite the large number of novel species, the Shannon diversity and Chao richness estimators are comparable to similar studies of mudflat microbial diversity in estuaries world-wide [39,40]. We have to keep in mind that the rarefaction curves show that with ~110 -135 clones the mudflats are under-sampled and that the actual richness and diversity are likely higher than estimated here especially since the Chao index is known to underestimate true richness at low sample sizes [41]. A more definite conclusion will require next generation sequencing approaches [37,38].

The major functional groups identified in this study and their relative contributions are common to many mudflat ecosystems [42,43]. The systems are driven by photosynthetic primary producers and there is an obvious important role for bacteria involved in sulfur cycling. Primary production is dominated by diatoms, whereas Cyanobacteria only play a minor role, given their low abundance. This is in agreement with the general prevalence of diatoms for fine grained muddy sediments, whereas Cyanobacteria favour sandy, course grained sediments [44]. Proteobacteria are the dominant players in sulfur cycling with sulfate reducing Deltaproteobacteria and sulfide oxidizing Gammaproteobacteria. Amongst the Deltaproteobacteria the sulfate reducers *Desulfobacteraceae* and *Desulfobulbaceae* dominate the population. *Desulfobulbaceae* are found in all sites while *Desulfobacteraceae* are absent from Damuan (with one exception) and Bandar. Apparently, the *Desulfobacteraceae* prefer the more saline mudflats and the mudflat from the most acidic region of the river in Bengkurong. Potentially there is a relationship with the presence of *Desulfobacteraceae* and the somewhat more extreme character of these mats relative to the Bandar and Damuan sites or alternatively, this class of organisms is more sensitive to the high anthropogenic carbon load and eutrophication at these two sites. Other potential sulfur (but not sulfate) reducing species were found amongst the Epsilonproteobacteria and mostly in Bengkurong (*Sulfurovum* and *Sulfurimonas)*. Sulfide oxidizing species were found amongst the Gammaproteobacteria, the most dominant bacterial class in all samples. These sulfide oxidizers belong to the *Chromatiales, Thiotrichales* and yet unclassified taxa with representatives *Thiohalophilus*, a typical marine sulfide oxidizing bacterium and *Candidatus Thiobios*, a symbiotic sulfide oxidizing organism that is found in symbiosis with marine ciliates [45]. Other members of the Gammaproteobacteria found in the Brunei mudflats have a heterotrophic lifestyle and are represented by the Xanthomonadales (mainly consisting of the uncultivated marine benthic group in the marine sites), Alteromonadales and Vibrionales, a large number of which are assigned as *Photobacterium* related species, typical marine species that are abundant in Pulau Pepatan. Representatives of the green sulfur bacteria, Chlorobi, are found in very low numbers, a single sequence in Bengkurong and Muara and 2 sequences in Pulau Pepatan. All four sequences were related to the anaerobic genus of *Ignavibacteria*. Alphaproteobacteria were found in all sites and mainly consist of the phototrophic bacteria belonging to the Rhodobacterales. Although initially believed not to oxidize sulfur, several Rhodobacterales species were shown to oxidize sulfur, sulfide or thiosulfate [46]. Bacteroidetes related species are found in high numbers in each sampled mudflat and are represented by the versatile heterotrophic members of the orders Cytophagales, Flavobacteriales and Sphingobacteriales that are involved in the degradation of a wide variety of complex organic compounds.

Cluster analysis of the phyla distribution as well as of the DGGE fingerprints gave a somewhat surprising result in that the mudflat at Bengkurong, which is exposed to acidic freshwater, appeared more similar to the marine and higher pH outlier mudflat of Pulau Pepatan. This result was independent of whether the chloroplast fraction was present or absent from the cluster analysis dataset. However, when looking at the proteobacterial fraction only, a clear distinction became visible between the three freshwater sites and the three marine sites. Potentially the conditions upstream of the Brunei River favour a lower contribution of diatoms and a larger contribution of Proteobacteria and Bacteroidetes similar to that on Pulau Pepatan. The cause of these effects cannot be determined at this moment, but requires an in depth study of the geochemical and microbial composition of the mudflats.

BES is situated within the biodiversity hotspot associated with the shallow South East Asian Sunda shelf that was above the sea level for much of the Quaternary glacial periods, Sundaland [47,48]. High biodiversity in this region is evident [49-51], but what drives this diversity is not well understood. One hypothesis for the high biodiversity in tropical regions in general, considers the potentially greater “effective” evolutionary time (evolutionary speed) as the result of shorter generation times, faster mutation rates, and faster selection at relatively stable and higher temperatures [52]. Distance decay as well as environmental factors appear important drivers of for example the diversity of nitrifying bacteria in salt marshes in temperate marshes as they also are for plants and animals [53]; these appear also important drivers in deep-sea bacterial communities in the china sea [54]. Analyses of literature further confirms that although present day environment are predominant drivers for bacterial communities, the historical component is always also present suggesting that bacterial communities like animals and plants are also determined by dispersal strategies and of similar distance decay [55]. Halfbeaks, freshwater fishes from Borneo, illustrate the geological evolution of diversity in Borneo: halfbeaks sampled from adjacent drainage basins but representing distinct paleo-drainages were phylogenetically dissimilar to one another, but were similar to halfbeaks from the same paleo-drainage on separate landmasses suggesting that geological drivers were important to seed landmasses such as the Malay Peninsula and Borneo before the Quaternary [56]. The mitochondrial DNA tree of the halfbeak genus *Dermogenys* is further consistent with radiation and speciation during the Quaternary, possibly before the last glacial cycle [56]. Yet geological history may be just one of many drivers leading to environmental heterogeneity which was demonstrated as a universal driver of species richness [57]. The uniqueness of the microbiome, as presented here for the BES, and previously shown for Sarawak forest soils [58], may be a key unexplored factor contributing to the botanical richness and species endemicity of Bornean environments [59].

## Conclusion

The Brunei River and Estuarine System harbours a unique microbial habitat with a high diversity of novel species that are of potential interest for metabolic and biotechnological exploration. Not only are the obtained sequences unique in comparison to other mudflats around the world, but there is also little overlap between individual site, with less than 10% of the sequences having more than 97% identity to known sequences in the Silva database. Despite this overwhelming number of novel species, the functional composition of these mudflats is well conserved in comparison to other mudflats. Given the extreme pH gradient over the river-estuary trajectory we expect more exciting discoveries in the water samples that are less stable than the sediment samples.

## Methods

### Sample sites and sampling

The Brunei River (Sungai Brunei, approximately 40 km long) is the shortest of three large river systems, including the Limbang and Temburong rivers, that feed into the estuarine system of the Inner Brunei Bay (IBB, North-west coast of Borneo, (4°57’40” N, 115°3’57” E), see Figure 1). The catchment area of the IBB is extensive (380 km^2^) and during fairly rare regional flooding (decadal), these rivers contribute to massive freshwater flux and carry high silt loads. Salinity and pH regimes along the BES are diurnally tidally-dominated by seawater fluxes, though during monsoon periods regular (daily) downpours shift these regimes along the BES and flatten their gradients [25,60]. Previously measured salinities vary between 0 and 34 psu, and pH varied between 5.7 and 8.4 units [25].

Sampling was undertaken along the length of the BES at six sites, from landward to seaward, as follows: Bengkurong, Damuan, Bandar, Sungai Besar, Muara, and Pulau Pepatan (Figure 1). Sampling coordinates are listed in Table 1. The mudflats around Pulau Pepatan (Pepatan Island), a mangrove covered island in the Brunei Bay that is not directly in the stream of the Sungai Brunei was chosen as an out-group.

Given diurnal tidal forcing and stochastically variable freshwater inflow, the frequency of exposure to estuarine water of high salinity/pH (which increases seaward) and of low salinity/pH (which increases landward) differs among the sites. Figure 2 shows the temporal variation in salinity and pH in the water at dedicated stations, Bandar (landward) and Keingarong, near Muara (extreme seaward). Salinity recordings were logged every 30 min between 8 and 30 Sept 2013, and both pH and salinity were recorded between 24 Oct and 01 Nov 2013, using HOBO conductivity data loggers (U25-001, Onset, Massachusetts, USA) and a WQL-pH logger (WTW GmbH, Germany). On site salinity and pH could not be accurately measured in the sediment, so were measured in the water flowing at approximately 1 meter from the sampling point; salinity was measured to ±0.01 psu (YSI Model 85D multimeter or HACH HQ40d meter and IntelliCal probe) and pH to ±0.01 units using an NBS scale (Thermo Orion Model 260A or HACH HQ40d calibrated with Mettler Toledo SRM NIST precision buffers). Additionally, the microbial communities at all of the sites are exposed to variation in pore-water pH in the sediments, which could counteract fluctuations in estuarine water pH (Hossain, unpublished data). Sample sites further differed in degree of siltation and organic loads. Anthropogenic and natural organic inputs are greatest around Damuan, and especially Bandar, via the Kedayang tributary (Marshall et al. 2008), which leads from the commercial centre of Brunei (Gadong and Kuilap). Although fringing mangroves along most of the Brunei River contribute natural organic material, treated and untreated sewerage is released near Bandar (Kampong Ayer and Pintu Malim, respectively). The sediment surfaces at Sungai Besar and Muara are inundated by increasing clearer, high saline waters, and are assumed less eutrophic. Furthermore, though the sand-size fraction of the sediments is generally higher at the seaward sites and finer-grained sediments (and a concomitant greater organic matter portion) are found landwards, sediment granulometry in the estuarine system is patchy [60].

Sampling was performed during the wet season on November 4^th^, 2012. Samples were taken at low tide when all mudflats were exposed to air and accessible. Samples were taken in triplicates using a sterile syringe corer, of which the top was removed. Approximately 10 mm of the top sediment layer was immediately mixed in lysis buffer containing tubes provided by the Ultra-clean DNA isolation kit (MO-BIO Laboratories, Inc., Carlsbad, CA, USA). Samples were initially stored at 4°C for a maximum of 3 hours and subsequently frozen at -20°C until nucleic acid extraction.

### Nucleic acid extraction

Total community DNA was isolated from approximately 1 g (wet weight) of sediment using the MO-BIO UltraClean Soil DNA Isolation Kit according to the manufacturer’s protocol for maximum yield. DNA concentrations were measured spectrophotometrically using the NanoDrop™ ND-1000 spectrophotometer (NanoDrop products, Wilmington, DE, USA). For controlled and reproducible downstream analysis the DNA concentration for each sample was adjusted to 15 ng/μl by adding diethylpyrocarbonate treated water.

### Ribosomal RNA gene amplification and sequencing

Bacterial 16S rRNA gene fragments were amplified from each sample by polymerase chain reaction (PCR) using the bacterial specific primers B8f (AGAGTTTGATCMTGGCTCAG) [61] and the universal primer U1492r (GGTTACCTTGTTACGACTT) [62]. A 50 μl of PCR mixture consisted of 200 μM of dNTPs (Roche Applied Science), 200 nM of each primer, 5% v/v of dimethyl sulfoxide (Sigma-Aldrich), 0.1% w/v of bovine serum albumin (Fermentas). 2 units of Hot Star Taq DNA polymerase (Qiagen Inc.) and 1 μl template. The reactions were run on a thermal cycler (Thermal Cycler 2720, Applied Biosystem) using the following PCR conditions: 15 min at 95°C; 35 cycles of 60 s at 95°C, 30 s at 53°, 120 s at 72°C and a final extension step for 7 min at 72°C. Amplicon sizes were verified by agarose gel electrophoresis. Amplicons of the desired size and concentration were purified on E.Z.N.A. Cycle Pure columns (Omega Bio-Tek Inc.) and cloned using the TOPO-TA cloning Kit (Invitrogen Corp.) following the manufacturer’s instructions. Positive transformants were identified by colony PCR using the M13 forward and reverse vector primers to amplify the inserted gene fragments. Amplicons containing insert DNA of the appropriate size were purified using Sephadex G-50 Superfine (Sigma-Aldrich) and DNA concentrations were determined spectrophotometrically. Amplicons were sequenced using the BigDye Terminator chemistry (Big Dye Terminator v3.1 Cycle Sequencing Kit, Applied Biosystem) according to the manufacturer’s instructions. The sequencing primer used was U1492r.

### Denaturing Gradient Gel Electrophoresis (DGGE)

Bacterial 16S rRNA gene specific DGGE-PCR reaction was performed using the following conditions. The PCR reaction mix contained per 25 μl of PCR mixture, 200 μM of dNTPs, 200 nM of each primer, 3% v/v of dimethyl sulfoxide, and 1 units of Taq DNA polymerase (GE Healthcare). The primers used were the universal U1492R primer and F968-GC (CGCCCGGGGCGCGCCCCGGGCGGGGCGGGGGCACGGGGGGCCTACGGGAGGCAGCAG) [63]. The PCR conditions were as following: initial denaturation for 3 minutes at 95°C, 10 cycles with denaturation for 60 s at 95°C, annealing for 60 s at 60°C/55°C (touch down with a 1°C decline per cycle) and an extension 120 s at 72°C, followed by 25 cycles of denaturation for 60 s at 95°C, annealing for 60 s at 55°C, extension for 120 s at 72°C and an final extension for 30 minutes at 72°C. Reaction mixtures were purified using E.Z.N.A. Cycle Pure columns. After purification, the DNA concentration in each sample was determined spectrophotometrically and adjusted to a final concentration of 200 ng of DNA in 28 μl, supplemented with 2 μl of loading buffer [64] and applied on the DGGE gel. The DGGE gel was run on the IngenyphorU® system (Ingeny International). A denaturing polyacrylamide gel (8% w/v acrylamide) was made according to the DGGE system manufacturer’s instructions using the gradient maker provided by the manufacturer in order to generate a urea-formamide gradient. The gradients ranged from 50-70% v/v. To 24 ml of the acrylamide-urea-formamide solution 50 μl 20% w/v ammonium-persulfate and 5 μl of tetramethylethylenediamine were added to initiate polymerization. The stacking gel consisting of 8% w/v acrylamide but lacking the denaturants was poured on top of the gel. Samples and reference samples were subjected to electrophoresis at 100 V for 18 h in a 0.5 strength TEA buffer at 60°C. After electrophoresis, DGGE gels were silver stained using an automated gel stainer (Hoefer Processor Plus, Amersham Biosciences). The following staining protocol was used; Gel fixation was achieved by soaking the gel for 30 min in a solution of 0.05% v/v acetic acid and 10% v/v ethanol. Gel staining was performed in a 0.2% w/v silver nitrate solution for 15 min, followed by three 1 min washing steps with Milli-Q water. After washing, the gels were processed for 5 min with a developing solution consisting of 1.5% w/v sodium hydroxide and 0.15% v/v formaldehyde. Finally, gels were soaked in 0.75% w/v sodium carbonate for 5 min to stop the developing and conserved by adding 10% v/v glycerine in 25% v/v ethanol and incubated for 7 min. For each step, 200 ml of the according solutions was used.

### Community fingerprint analysis

The DGGE fingerprints were analysed using the BioNumerics software package (Applied Maths NV, Sint-Martens-Latem, Belgium). Fingerprints were normalized, subjected to the curve based Pearson correlation method to create the similarity index and clustered using the UPGMA algorithm.

### DNA sequence and phylogenetic analysis

Geneious software (Geneious version (R6: 6.1.8) created by Biomatters. Available from http://www.geneious.com) was used to analyse and correct the ABI trace files and to conduct phylogenetic analysis. 16S rRNA Sequences were compared to two different databases in an offline Blast integrated in the Geneious software. The SILVA SSU Ref database is a cured database of 16S rRNA sequences obtained from isolated species with a minimal length of 1200 bases [65]. The non-redundant version of the SILVA SSU Ref database (Silva SSU ref NR) contains sequences derived from isolated species but also from a large number of uncultivated species obtained via environmental studies. The best blast hits were used for phylogenetic annotation at several taxonomic levels. General statistics on operational taxonomic units (OTU’s) and estimation of richness and diversity were performed on the collected fasta files using the molecular analysis software package Mothur [66]. The sequences were checked for potential chimeric sequences using the online chimera check tool Decipher (http://decipher.cee.wisc.edu/FindChimerasOutputs.html). Suspected chimera were manually checked and removed if required.

DNA sequences described in this study were deposited in GenBank with the accession numbers KJ941391 - KJ942135.

## Competing interests

The authors declare that they have no competing interests.

## Authors’ contributions

HB, HS and DJM were involved in the field work campaign. Molecular analysis was carried out by HB, HS and JK. All authors were involved in drafting the manuscript. All authors read and approved the final manuscript.
